# Oblique Axis Rib Stretch and Curved Planar Reformats in Patients for Rib Fracture Detection and Characterization: Feasibility and Clinical Application

**DOI:** 10.1155/2023/4904844

**Published:** 2023-08-29

**Authors:** Jingzhi Ye, Hongyi Li, Meng Zhang, Fenghuan Lin, Jingfeng Liu, Jun Chen, Ye Peng, Mengqiang Xiao

**Affiliations:** ^1^Department of Radiology, Zhuhai Hospital, Guangdong Provincial Hospital of Traditional Chinese Medicine, 53 Jingle Road, Zhuhai City, Guangdong Province, China; ^2^The Second People's Hospital of Xiangzhou District, 21 Nanquan Road, Zhuhai City, Guangdong Province, China

## Abstract

**Objective:**

To assess the use of CT with oblique axis rib stretch (OARS) and curved planar reformats (CPRs) for rib fracture detection and characterization.

**Methods:**

A total of 108 forensically diagnosed patients with rib fractures were evaluated retrospectively. OARS and CPRs were independently used during the diagnosis in two groups. In each group, the final diagnosis was made after a junior radiologist's initial diagnosis was reviewed by a senior radiologist. The images were evaluated for the presence and characterization of rib fractures.

**Results:**

A total of 2,592 ribs were analyzed, and 326 fractured ribs and 345 fracture sites were diagnosed using reference standard. Two groups of radiologists identified 331 and 333 fracture sites using the OARS method, 291 and 288 fracture sites using the CPRs method, and 274 fracture sites in forensically diagnosed patients (CR: conventional reconstruction), respectively; and all missed diagnoses were nondisplaced rib fractures. The ROC A_z_ value of OARS_1,2_ was 0.98, which is higher than CPRs_1,2_ 0.91, and CR 0.90 (all *p* < 0.01). The A_z_ value for detecting rib fractures using CPRs_1,2_ and CR has no statistical difference (*p* = 0.14 and 0.29). More misdiagnosed patients were found using CPRs_1,2_ (42 and 44 cases) than OARS_1,2_ (1 and 2 cases) and CR (2 cases). The displaced fracture detection ratio of all methods showed no difference.

**Conclusions:**

Doctors using the OARS method could improve diagnostic performance for detecting rib fractures without the requirement of specialized software and workstation when compared with CPRs.

## 1. Introduction

Traumatic rib fractures represent the most common injury sustained following thoracic trauma [1]. They occur in about 10% of patients, of whom 94% have additional injuries, of which 12% are lethal [2]. Rib fractures account for approximately 10% of all trauma admissions and are seen in up to 39% of patients sustaining blunt thoracic trauma [3–5]. Young patients often suffer rib fractures after high-energy trauma such as a motor vehicle collision or a fall from height [6]. The number of rib fractures, pre-existent pulmonary pathology, and age are known risk factors for rib fracture-associated mortality and morbidity [5, 7]. Rib fractures can be of various types (unicortical, bicortical, hair-like, and trabecular only). However, the most clinically relevant factor is whether the fracture is displaced or not. The classification of fractures into displaced and nondisplaced fractures is essential as it provides valuable information about the type of fracture and the available treatment options [7, 8]. Nondisplaced fractures are characterized by the absence of angulation or shortening, a fracture line width of less than 2 mm, and/or less than 1 mm displacement of the bone cortex. On the other hand, displaced fractures are identified by a fracture line width of more than 2 mm and/or more than 1 mm displacement of the bone cortex [9, 10]. Ribs are long arched bones with a posterior cylindrical shape and a flatter anterior shape. Ribs have a downward obliquity which is a variable depending on various factors (e.g., rib level, patient sex, size, and age) [11, 12]. This anatomy makes rib cage CT evaluation a meticulous and time-consuming process because no orthogonal plane is optimal for visualization of all the ribs of an individual, undoubtedly, identifying rib fractures is time-consuming, and no dedicated standardized rib cage reformation is routinely used for the detection of rib fractures.

Recently, CPRs improved the sensitivity and specificity of diagnosing rib fractures and shortened the diagnostic time [13, 14]. Although various methods were used to improve the accuracy of rib fracture diagnosis, such as CPRs [13, 14] and automated rib fracture detection software [15, 16], they were difficult to generalize because these methods require specialized software and workstations. In the process of developing a deep learning computer-aided diagnosis system for rib fractures, based on the labeling of 1563 cases of rib fractures, we created oblique axis rib stretch (OARS), which could display the anterior and lateral segment, posterior segment of each rib in the same plane according to the rib shape, and parallel rib reconstruction; the whole rib can be visualized in the same plane in some patients, and we were able to accurately perform the diagnosis of rib fracture. The primary aim of this study was to compare OARS, CPRs, and CR CT images for rib fractures' detection and characterization.

## 2. Materials and Methods

### 2.1. Patients

The Institutional Review Committee of our hospital approved this retrospective study and exempted the requirement of informed consent (BF2020-323). All work-related injuries and traffic accidents which require judicial identification of patients in Zhuhai Hospital, Guangdong Provincial Hospital of Traditional Chinese Medicine, from 2019 to 2021 were collected. To achieve accuracy, work injury appraisal has to use CT for diagnosis in our hospital. A junior radiologist makes the initial diagnosis, and a senior radiologist with more than 10 years' experience review for the initial diagnosis and make the final forensic diagnosis with the CT conventional reconstruction (CR) images. For our study, 138 patients were collected initially. Inclusion criteria were as follows: patients aged ≥18 years. Exclusion criteria were as follows: (a) no rib fractures were found in patients, (b) respiratory motion artifact affects the image observation, and (c) those with pathological rib fractures. Five patients under age 18 and five patients affected by the respiratory motion artifact were excluded. Eight patients finally found no rib fractures were also excluded. Finally, 108 patients were included in our study (mean age 52.43 ± 11.18 year; male = 80, female = 28).

### 2.2. Scan Methods and Technique Details

All scans were performed on Canon 320-row detector CT (Aquilion One Vision, Canon Medical Systems, Otawara, Japan). The scanning parameters were set as follows: tube voltage of 120 kV, tube currents of 100 mA–150 mA, slice thickness 0.5 mm, interval thickness 0.5 mm, and FOV 512 ∗ 512. All images were reconstructed with the adaptive iteration reconstruction technique (AIDR 3D standard; Canon Medical Systems). All CT scans were reviewed by a radiologist with three-dimensional reconstructions (conventional reconstruction) as needed (Canon workstation, Canon Medical Systems, Otawara, Japan).

### 2.3. OARS

**Figure 1:**
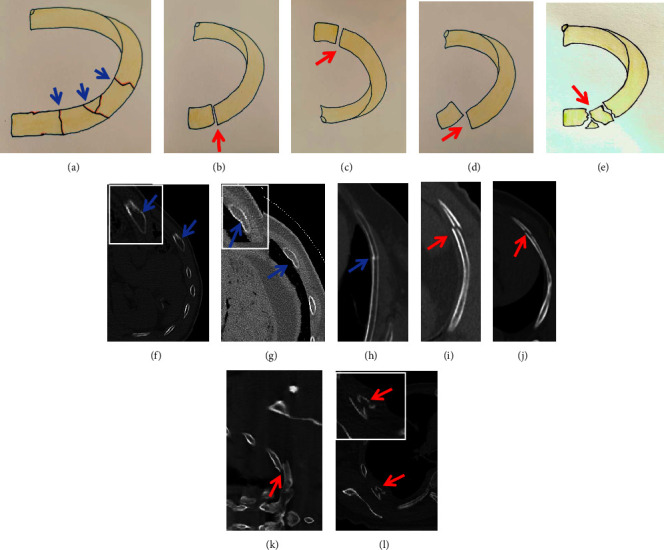
Hand-drawn graphics indicate nondisplaced fractures ((a) blue arrow) and depict different types of displaced fractures ((b)–(e) red arrow). In Figures 1(f)–1(h), nondisplaced fractures are shown (blue arrow). Figures 1(b) and 1(i) exhibit displaced fractures with more than 1mm displacement of the bone cortex (red arrow). Figures 1(c) and 1(j) demonstrate a displaced fracture with more than 2mm displacement of the bone cortex (red arrow). Figures 1(d) and 1(k) show a clearly displaced fracture with complete displacement of margins of the cortex. Figures 1(e) and 1(l) demonstrate comminuted fracture, which is a special case of displaced fracture with three or more fragments (red arrow).

**Figure 2:**
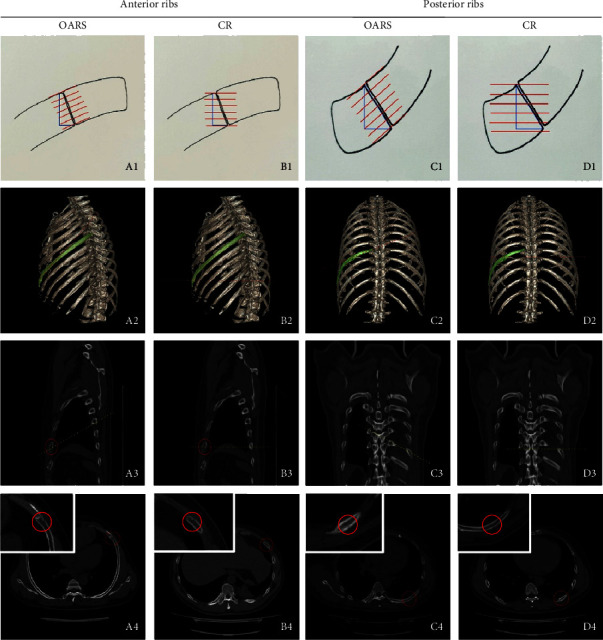
Hand-drawn graphics (A1–D1) showed the reconstruction orientation of OARS (A1, C1) and CR (B1, D1) of anterior or posterior limbs: more fracture lines can be shown when using the vertical fracture line reconstruction method (OARS). Orientation (red lines or yellow lines) of OARS and CR of anterior or posterior limbs on 3D images (A2–D2) and sagittal (or coronal) images (A3–D3). Anterior or posterior limbs' fractures (red circles) displayed on axial images using OARS (A4, C4) and CR (B4, D4). OARS: oblique axis rib stretch. CR: conventional reconstruction.

Original DICOM images were uploaded to the PACS system (Yi Lianzhong, Xiamen City, Fujian Province, slice gap and slice thickness were 0.5 mm). Areas outside the field of interest were discarded (e.g., head, pelvis, and legs) (see Figure1). The OARS method includes two steps: (a) anterior and lateral segment reconstruction (Table 1, Figure 2) and (b) posterior segment reconstruction (Table 1, Figure 2). The thorax was divided into 3 equal parts, with the ribs divided into anterior, lateral, or posterior according to the position [15].

### 2.4. CPRs

**Figure 3:**
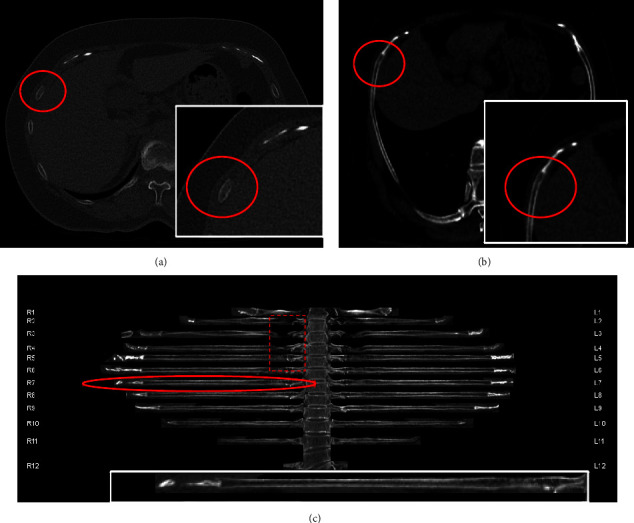
A 59-year-old female patient was diagnosed with multiple fractures, including a fracture of the 7th anterior rib as indicated on the OARS image (b). Interestingly, none of the radiologists were able to detect the fracture (cortical folds) on either the CR image (a) or the CPRs' image (c). It should be noted that the chest rib joint area in the CPRs' image (c) is prone to misdiagnosed fractures (indicated by the rectangular dashed line). Within this region, fractures were identified in the right 6th, 7th, and 8th posterior ribs, as well as the left 6th and 8th posterior ribs, while the left 5th posterior rib was mistakenly diagnosed as a fracture. OARS: oblique axis rib stretch. CR: conventional reconstruction. CPRs: curved planar reformats.

Original DICOM images were uploaded to Siemens workstation (syngo.via, version: VB10A; workflow: Bone Reading, Siemens Healthcare GmbH). After automated segmentation, a spider-like image was generated with the vertebral column as the body and the 24 ribs as perpendicular extremities (Figure 3). The vertebrae and the ribs of each side were labeled with numbers from 1 to 12. The labels were constantly displayed next to the ribs. The single image featuring in-plane reformation of the rib cage was obtained for each of the 108 patients from the 0.5 mm slice thickness data. These reformations were performed automatically in a postprocessing step and visualized by the software. In our study, no reformation images need to be excluded because of poor image quality. All images were observed in Siemens workstation by the radiologists.

### 2.5. Image Assessment and Statistical Analysis

The injury appraisal diagnosis results were regarded as the diagnosis result using conventional reconstruction (CR, the results of our hospital diagnostic report); CR: the analysis was based on all images available in the PACS, standard axial view, sagittal view, coronal view (layer thickness, layer spacing 2 mm), 3D rib reconstruction, thin layer axial view (layer thickness, layer spacing 0.5 mm), and thin layer images can be used for multiplane reconstruction. In addition, radiologists (Group 1: two radiologists, one junior and one senior, with three and fifteen years of CT analysis experience, respectively. Group 2: two radiologists, one junior and one senior, with four and twelve years of CT analysis experience, respectively) reviewed all the CT examinations with OARS and CPRs independently, while all injury appraisal diagnosis results were hidden. The final diagnosis was made after a junior radiologist's initial diagnosis was reviewed by a senior radiologist. Observers were allowed to adjust image brightness and contrast for simulating the routine clinical interpretation environment. All ribs were evaluated for the presence, type (displaced or nondisplaced fracture, where one rib is fractured in two or more places), and location (anterior, lateral, or posterior) of rib fractures. The radiologists also documented the displacement directions (inward/outward or upward/downward). For rib fractures without displacement, they recorded the sites of cortical fracture (medial/lateral margin or superior/inferior margin of bone cortex).

### 2.6. Reference Standard

Another two senior radiologists with 20 and 18 years of experience in CT diagnosis established the standard of reference in consensus. To achieve the best possible standard of reference in this study, the radiologists were allowed to use CPRs and OARS, respectively, as well as all results and drawings from all four readers of all patients, then have a consensus adjudication session for discrepant readings. For each fracture found, a unique identifier number was assigned in the standard of reference. This number was calculated from the patient number and the number code of the location (derived from the affected number of ribs and side). The diagnostic criteria for rib fractures included visualization of the fracture line with or without displacement.

### 2.7. Statistical Analysis

Statistical analysis was conducted using IBM SPSS Statistics (version 26.0, IBM Corp, Armonk, NY, USA). For normally distributed quantitative data, results are presented as mean ± standard deviation (SD). For non-normally distributed quantitative data, results are expressed as median (interquartile range) or as median (upper limit and lower limit). Sensitivity, specificity, positive predictive value (PPV), negative predictive value (NPV), and diagnostic accuracy for detecting rib fractures were calculated. Differences in sensitivity, specificity, PPV, NPV, and accuracy among the groups were assessed using the chi-square test. The observer performance for each method was evaluated by calculating the area under the receiver operating characteristic (ROC) curve (AUC). Statistical significance was set at a 2-tailed *p* value of less than 0.05.

## 3. Results

A total of 108 patients were diagnosed with single or multiple fractures. A total of 2,592 ribs were analyzed; 326 ribs and 345 fracture sites were found by reference standard (19 ribs presented multiple fractures): 106 rib fractures were considered to be displaced and 239 rib fractures were nondisplaced. Fracture locations were as follows: 193 anterior, 71 lateral, and 81 posterior. For 239 nondisplaced rib cortical fracture, 87.45% (209/239) were of medial/lateral margin, 2.51% (6/239) were of superior/inferior margin, and 10.04% (24/239) were of all four margins. For displaced fractures, 41.51% (44/106) rib displaced inward/outward, 1.89% (2/106) displaced upward/downward, and 56.60% (60/106) displaced in all directions.

**Figure 4:**
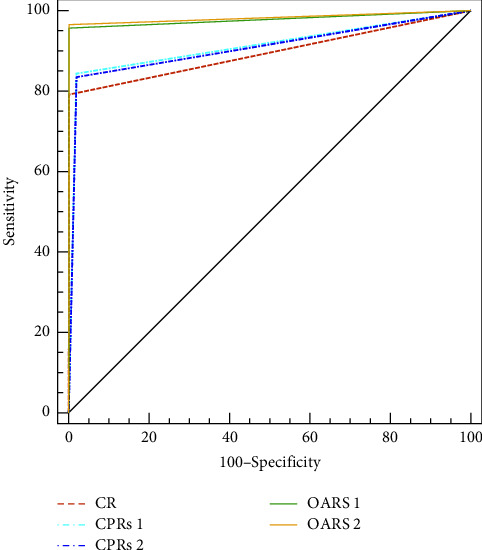
Comparison of areas under the curve (AUC) for receiver operating characteristic curves of OARS, CPRs, and CR (OARS_1_: 0.98 (95%CI: 0.97–0.98); OARS_2_: 0.98 (95%CI: 0.98–0.99); CPRs_1_: 0.91 (95%CI: 0.90–0.92), CPRs_2_: 0.91 (95%CI: 0.90–0.92); CR: 0.90 (95%CI: 0.88–0.91. OARS: oblique axis rib stretch. CPRs: curved planar reformats. CR: conventional reconstruction.

The diagnostic performance with different reconstruction methods in each group is shown in Table 1. The pairwise comparison results showed the following: OARS_1_ and OARS_2_ exhibited higher sensitivity than the other two reconstruction methods (*p* < 0.001). There was no statistically significant difference in sensitivity between CPRs_1_ and CPRs_2_ compared to the CR method (*p* = 0.06, 0.09). The PPV of CPRs_1_ and CPRs_2_ was lower than that of the CR method and OARS_1_ and OARS_2_ (*p* < 0.001). There was no statistically significant difference in PPV between CPRs_1_ and CPRs_2_ compared to OARS_1_ and OARS_2_ (*p* > 0.10). There were no statistically significant differences in specificity, NPV, and accuracy among the different groups (*p* > 0.28). In summary, the OARS method has the best diagnostic performance of any method, with no significant differences from the gold standard. To a certain extent, there may be a difference in diagnostic sensitivity between the CPR and CR methods, but this difference was not statistically significant. However, the diagnostic accuracy showed no significant difference with forensic diagnosis results (CR). The A_z_ value for detecting rib fractures using OARS (OARS_1_: 0.98 (95% CI: 0.97–0.98); OARS_2_: 0.98 (95% CI: 0.98–0.99)) was higher than CPRs (CPRs_1_: 0.91 (95% CI: 0.90–0.92), all *p* < 0.01; CPRs_2_: 0.91 (95% CI: 0.90–0.92), all *p* < 0.01) and CR (0.90 (95% CI: 0.88–0.91), all *p* < 0.01). There was no statistical difference in the A_z_ value for detecting rib fractures using CPRs_1,2_ and CR (*p* = 0.14 and 0.29) (Figure 4).

More specifically, the missed diagnosis rate for nondisplaced rib fractures was 5.86% in OARS_1_ and 5.08% in OARS_2_, followed by 22.88% in CPRs_1_, 23.85% in CPRs_2_, and the highest rate of 29.71% in CR (Figure 2). All of the missed diagnoses were nondisplaced rib fractures. More misdiagnosed were found using CPRs_1,2_ (42 and 44 cases) than OARS_1,2_ (1 and 2 cases) and CR (2 cases). Readers misdiagnosed 18 cases and 16 cases of displaced fractures as nondisplaced fractures in CPRs_1,2_; 6 cases and 3 cases in OARS_1,2_ and 1 case during work injury appraisal (conventional reconstruction), respectively. Location-based rib fractures diagnostic accuracy were described as follows: 6 cases and 9 cases of anterior were misdiagnosed as lateral segment in CPRs_1,2_, respectively, while 1 case of the lateral segment was misdiagnosed as anterior segment in CPRs_1_(Figure 4). In OARS_1,2_, 4 cases and 5 cases of lateral rib fractures were misdiagnosed as posterior segment, while 2 cases and 3 cases of lateral were misdiagnosed as anterior segment, respectively. No location-based misdiagnoses were found during work injury appraisal.

## 4. Discussion

Rib fractures are the commonest form of bone injury after blunt thoracic trauma and can be potentially life-threatening. Correct diagnosis of rib fractures with a comprehensive display of rib fractures is of forensic and clinical significance. Rib fractures can be easily detected on conventional plain chest radiography when the rib fractures are displaced type [13]. Furthermore, missed diagnosis and inconsistency are common, especially after a long day at work [17]. The sensitivity of radiologists with detecting displaced rib fractures with different methods was 100%. The missed diagnoses in our study were all nondisplaced fractures. As demonstrated by this work, using OARS reformation improves diagnostic performance for the detection of rib fractures, which is better than CPRs and CR. CPRs reformation have the same diagnostic effect as CR.

As was already mentioned, accurate diagnosis of rib fractures is crucial for forensic identification of work injuries. The subsequent identification decision is negatively impacted by misdiagnosis and missed diagnoses from imaging, which may result in lower (higher) workers' and traffic accident compensation. In addition, missed diagnoses, especially of nondisplaced rib fractures, can lead to inadequate pain management for patients. Such oversights can precipitate complications, including lung collapse, which can profoundly impact a patient's quality of life. It is noteworthy that the first rib is anatomically proximate to the vertebral and subclavian arteries. Studies have shown that the incidence of vascular injury accompanying a nondisplaced fracture of the first rib is 3% [18]. Furthermore, treating a combination of both displaced and nondisplaced fractures in a single patient presents greater complexities than addressing solely displaced fractures [19].

To the best of our knowledge, we are the first to study the classification of the rib cortex and to count the types of rib fracture cortex. The nondisplaced fracture sites in this study were as follows: 209 medial and lateral cortical fractures, 6 superior and inferior cortical fractures, and 24 cortical fractures in all directions. The displaced fracture sites in this study are as follows: 44 outward or inward displacement, 2 upward and downward displacement, and 60 displacement containing both outward-inward and upward-downward. The medial and lateral bone cortical fractures (whether displaced or not) accounted for 97.97% (338/345) of all fractures. The occurrence of a substantial number of medial and lateral fractures can shed light on the increased susceptibility of rib fractures to cause damage to critical organs such as the lungs, liver, spleen, and others. This observation served as a motivation for establishing the OARS method. It is widely recognized that the vertical fracture line reconstruction method provides the most effective approach for fracture observation from CT images.

Our team used OARS reconstruction according to the anatomical characteristics of the steeper shape of the anterior and lateral rib segment and the straighter course of the posterior rib segment [11, 12]. OARS step A reconstruction (Figure 2, A1–A4) could display the anterior rib and lateral rib: 68.63% of the anterior rib and lateral rib are displayed on the same plane, and about 31.37% of the anterior rib, lateral rib, and posterior rib, that is, one complete rib can be displayed on the same plane. OARS step B (Figure 2, C1–C4) shows the posterior rib, and 99.6% of the posterior rib can be clearly displayed on the same plane. In our study, the anterior and lateral rib fractures accounted for 76.52% (193 + 71 = 264,264/345) of all rib fractures, and the posterior rib accounted for 23.48% (81/345). Each slice of the OARS images was oriented perpendicular to the fracture line (medial and lateral bone cortical fractures). Because ribs are long arched bones with a longer superior-inferior distance and a shorter anterior-posterior distance, the OARS method is advantageous for visualizing the fracture line and interpreting the displacement (Figure 2, A1–D1). Radiologists using OARS reconstruction to diagnose rib fractures were better than CPRs and CR reconstruction. In terms of fracture positioning accuracy, there were 6 cases of positioning errors in junior radiologists and no case was found in senior radiologists. However, it is relatively difficult to accurately locate the anterior, lateral, and posterior ribs with CPRs or CR method. When radiologists applied the CPRs method, there were 6 and 9 cases of rib fracture location errors by CPRs_1,2_, respectively, and the location of fracture affected the prognosis of patients [8, 20].

Reconstruction can be done automatically with CPRs, but additional software and workstation is required, and it takes some time to upload the original image for reconstruction; when reading the CPRs images, the protrusion of physiologic focal changes in rib contour (small protrusions or depressions in the cortical bone) may be misdiagnosed as rib fractures (Figure 3). In addition, some medial and lateral cortical fractures ribs (nondisplaced) could be missed. Two cases of costal cartilage fracture in CPRs were all missed diagnosis in our study (Figure 3). The diagnostic performance of CPRs in this study was consistent with the Dankerl, P's research [12], and there was no improvement in the diagnosis of fractured ribs in our study.

Previous study showed that the most common missed diagnosis by the CR method was anterior segment rib fracture, accounting for 55.2% of all missed diagnosis [20]. In this study, anterior rib fractures had the highest missed diagnosis rate, accounting for 59.15% (193−151 = 42, 42/71) of all missed fractures. We speculated that the cause of the missed diagnosis is related to the anatomical morphology of the oblique inward and downward walking of the anterior ribs, which can show less slices of the fracture than the lateral ribs at the same slice thickness and slice gap (Figure 2). CR has the lowest diagnostic sensitivity of approximately 79.42% among three methods, which is consistent with other studies that obtained a sensitivity of 46.3%–80.8% for routine reconstruction in diagnosing fractures [12, 21]. The missed diagnosis rate of rib fractures with the CR method in this study was 20.58%, which was consistent with the previous study [10]. There was controversy about the diagnostic sensitivity of CPRs versus CR methods: one previous study showed that the diagnostic sensitivity of CR was higher than CPRs [22], while others showed the opposite results [11, 12]. In this study, the sensitivity of CPRs reconstruction was slightly but not statistically significantly higher than that of CR.

This study has limitations. First, due to its retrospective design, the rib fractures were not directly clinically matched to the patient symptoms; therefore, the proportion of minor injured patients may be larger than that of patients with severe injuries, and missed rib fractures may be found more frequently in patients with minor injuries. Second, this study was conducted in a single center. The quality of OARS and CPRs reconstructed images was related to the CT scanning parameters. However, scanners from other vendors may offer lower image quality using the same scanning parameter settings with different materials of the tube or detector and iterative reconstruction algorithm.

## 5. Conclusions

OARS demonstrated a notably superior diagnostic sensitivity and accuracy in detecting rib fractures compared to CPRs methods. While CPRs and CR exhibited comparable diagnostic performances to each other, both were inferior to OARS. In addition, the advantage of the OARS method is that it enhances diagnostic performance without the need for specialized software and workstations.

## Figures and Tables

**Table 1:** The number of fractures detected in each site and diagnostic performance with different reconstruction methods of each group.

Group	Anterior	Lateral	Posterior	Sensitivity (%)	Specificity (%)	PPV (%)	NPV (%)	Accuracy (%)
OARS_1_^#^	184	69	78	95.94	99.96	99.70	99.38	99.46
OARS_2_^#^	184	70	79	96.52	99.91	99.40	99.47	99.54
CPRs_1_	153^*∗*^	70	68^*∗*^	84.35^*∗*^	98.17	87.39^*∗*^	97.65	97.92
CPRs_2_	148^*∗*^	70	70^*∗*^	83.48^*∗*^	98.08	86.75^*∗*^	97.53	97.80
CR	150^*∗*^	55^*∗*^	69^*∗*^	79.42^*∗*^	99.91	99.28	96.94	97.26

OARS: oblique axis rib stretch; CPRs: curved planar reformats; CR: conventional reconstruction. ^*∗*^*p* < 0.05, when compared with the gold standard. ^#^A total of 813 and 815 whole ribs (anterior, lateral, and posterior) are shown on the same CT images (about 31.37 percent of all ribs) could be displayed using OARS A step in groups 1 and 2, 1776 and 1763 ribs (anterior and lateral) are shown on the same CT images using OARS A step in groups 1 and 2, respectively. OARS B step can be used to display almost every posterior rib (2588 and 2586 posteriors ribs in groups 1 and 2, respectively), which makes up 99.86% of all ribs. OARS A + B steps can show each complete rib.

## Data Availability

The datasets used or analyzed during the current study are available from the corresponding author on reasonable request.
